# Chalcone-Induced Apoptosis through Caspase-Dependent Intrinsic Pathways in Human Hepatocellular Carcinoma Cells

**DOI:** 10.3390/ijms17020260

**Published:** 2016-02-22

**Authors:** Rodrigo Ramirez-Tagle, Carlos A. Escobar, Valentina Romero, Ignacio Montorfano, Ricardo Armisén, Vincenzo Borgna, Emanuel Jeldes, Luis Pizarro, Felipe Simon, Cesar Echeverria

**Affiliations:** 1 Laboratorio de Bionanotecnologia, Universidad Bernardo O Higgins, General Gana 1780, Santiago 8370854, Chile; rramirez@ubo.cl (R.R.-T.); valentina.romero@ubo.cl (V.R.); montorfano3320@gmail.com (I.M.); 2 Departamento de Ciencias Químicas, Laboratorio de Síntesis Orgánica, Universidad Andres Bello, Av. República 275, Santiago 8370146, Chile; cescobar@unab.cl; 3 Centro de Investigación y Tratamiento del Cancer, Facultad de Medicina, Universidad de Chile, Santiago 8380453, Chile; ricardoarmisen@med.uchile.cl; 4 Center for Excellence in Precision Medicine Pfizer, Pfizer Chile, Obispo Arturo Espinoza Campos 2526, Macul, Santiago 7810305, Chile; 5 Urology Department, Hospital Barros Luco Trudeau, San Miguel, Santiago 8900085, Chile; vborgna@gmail.com; 6 Andes Biotechnologies SA and Fundación Ciencia para la Vida, Zañartu 1482, Ñuñoa, Santiago 7780272, Chile; e.jeldes1@gmail.com; 7 Department of Biological Science, Faculty of Biological Science, Universidad Andrés Bello, Santiago 8370146, Chile; 8 Instituto Nacional del Cancer, Universidad de Chile, Profesor Zañartu 1010, Santiago 8380455, Chile; drluispizarro@gmail.com; 9 Laboratorio de Fisiopatología Integrativa, Departamento de Ciencias Biologicas, Facultad de Ciencias Biologicas and Facultad de Medicina, Universidad Andres Bello, Avenida Republica 239, Santiago 8370146, Chile; fsimon@unab.cl; 10 Millennium Institute on Immunology and Immunotherapy, Santiago 8331150, Chile

**Keywords:** Chalcone, Caspase, Reactive Oxygen Species

## Abstract

Hepatocellular carcinoma (HCC) is one of the most commonly diagnosed cancers worldwide. Chemoprevention of HCC can be achieved through the use of natural or synthetic compounds that reverse, suppress or prevent the development of cancer progression. In this study, we investigated the antiproliferative effects and the mechanism of action of two compounds, 2,3,4′-trimethoxy-2′-hydroxy-chalcone (CH1) and 3′-bromo-3,4-dimethoxy-chalcone (CH2), over human hepatoma cells (HepG2 and Huh-7) and cultured mouse hepatocytes (HepM). Cytotoxic effects were observed over the HepG2 and Huh-7, and no effects were observed over the HepM. For HepG2 cells, treated separately with each chalcone, typical apoptotic laddering and nuclear condensation were observed. Additionally, the caspases and Bcl-2 family proteins activation by using Western blotting and immunocytochemistry were studied. Caspase-8 was not activated, but caspase-3 and -9 were both activated by chalcones in HepG2 cells. Chalcones also induced reactive oxygen species (ROS) accumulation after 4, 8 and 24 h of treatment in HepG2 cells. These results suggest that apoptosis in HepG2 was induced through: (i) a caspase-dependent intrinsic pathway; and (ii) by alterations in the cellular levels of Bcl-2 family proteins, and also, that the chalcone moiety could be a potent candidate as novel anticancer agents acting on human hepatomas.

## 1. Introduction

Hepatocellular carcinoma (HCC) is one of the most commonly occurring cancers in the world [1]. The incidence of HCC in Asia and Africa countries is 120 per 100,000; however, of 4 to 15 per 100,000 has been reported in Western countries [2]. Unfortunately, HCC is a relatively chemotherapy-resistant tumor mainly due to their heterogeneity and because of multidrug resistance phenotype’s development [3]. Thus, current treatments available for liver cancer treatment remain unsatisfactory [4]. Apoptosis resistance, a hallmark of some human cancers, is tightly linked to the clinical cancer therapy failure, such as chemotherapy and radiotherapy [5,6]. Hepatocellular carcinoma (HCC) is one of the few cancers with well-defined major risk factors [7,8]. In 80% of cases, HCC develops in a cirrhotic liver, and cirrhosis is the strongest predisposing factor [7]. Genotoxic stress-induced apoptotic cell death by chemotherapy remains the primary anticancer treatment. However, despite the tremendous strides made in the development of targeted anticancer therapies, the prevalence of resistance to genotoxic drugs is still a major obstacle in the successful management of resistant and aggressive tumors [9,10,11]. Several studies have suggested a potential contribution of the reactive oxygen species (ROS) to oncogenesis and resistance to antitumor treatments [12,13]. Oxidative stress may also contribute to cancer initiation through ROS-induced DNA damage [14]. 

Conventional therapies for the treatment of hepatocarcinoma include, among others, chemotherapy, radiation, and surgical resection. Ablation fails to restore health because of insufficient diagnosis and serious side effects. Recently, an additional therapy has been introduced based on the application of antioxidants to decrease ROS-induced injury and to improve immunity function in rats suffering HCC [15,16]. Therefore, the development of more effective and less toxic chemoprevention agents is necessary to prevent or retard the process of hepatocarcinogenesis [17].

Chalcones (1,3-diaryl-2-propen-1-ones) are precursors in the biosynthesis of flavonoids/isoflavonoids. Chemically, a chalcone consists of an open-chain flavonoid with two aromatic rings joined by a three-carbon α,β-unsaturated carbonyl system [18,19]. Chalcones are reported to possess anti-inflammatory, antimicrobial, antioxidant and anticancer properties [19,20,21,22,23] and are components found spread in vegetables, and accessible to human consumption through a diet rich in fruits and vegetables.

The aim of this work is to determine the apoptotic and/or cytotoxic capabilities of two compounds, 2,3,4′-trimethoxy-2′-hydroxy-chalcone (CH1) and 3′-bromo-3,4-dimethoxy-chalcone (CH2), when acting over human hepatoma cells line (HepG2 and Huh-7). The present study also investigated the expression of caspase-8, caspase-9, caspase-3 and alterations in the cellular levels of Bcl-2 family proteins in HepG2 cells as well as the induction of ROS. Our results indicate that the two chalcones mediate the apoptosis in HepG2 cells in an intrinsic, caspase-dependent pathway, but also that they increase the presence of ROS. In contrast, they have no cytotoxic effects in cultivated primary mouse hepatocytes.

## 2. Results

### 2.1. Cytotoxic Effect of Chalcones in Human Hepatoma Cells (HepG2) and Normal Mouse Hepatocytes (HepM)

**Figure 1:**
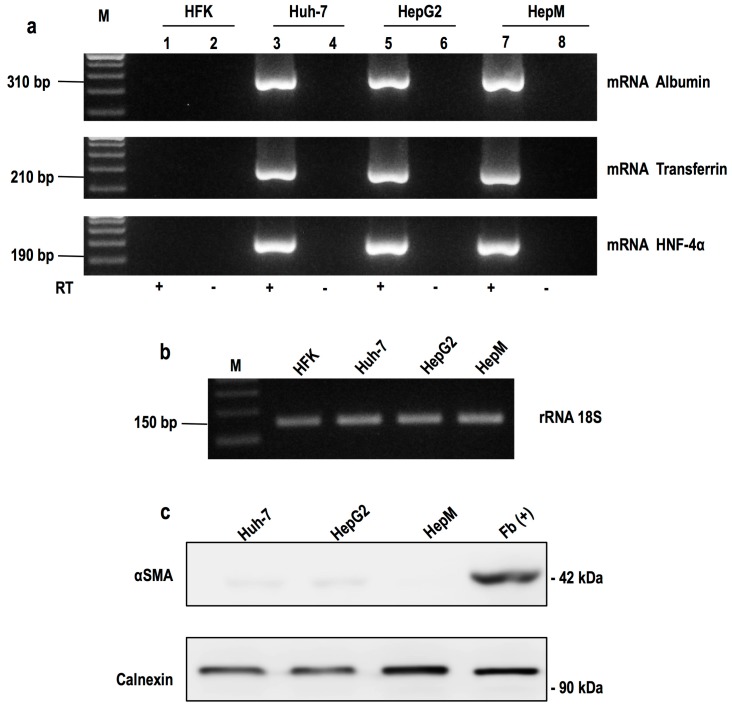
Expression of hepatic markers by RT-PCR and Western blot: (**a**) Markers of hepatic mRNAs were amplified. Electrophoresis agarose seen in the amplified fragments of 310, 210 and 190 bp, corresponding to albumin, transferrin and HNF-4α, respectively. The fragment was generated from the cDNA obtained from total RNA Huh-7, HepG2 and HEPM cells. Huh-7 and HepG2 cells were used as positive control. HFK cells were used as negative control. Reverse transcriptase in the absence of product is not detected (lanes 2, 4, 6, 8). M corresponds to the marking of 100 bp molecular size; (**b**) Constitutively expressed gene 18S rRNA was used as an internal control; (**c**) Expression of the protein αSMA. By Western blot, the protein expression of αSMA was determined in the hepatic (Huh-7, HepG2 and HepM) and fibroblast cells (Fb (+)). Constitutively expressed calnexin was used as an internal control.

Once the primary culture of mouse hepatocytes (HepM) were obtained, we proceeded to confirm the culture purity by reverse transcription polymerase chain reaction (RT-PCR) and Western blotting. The mRNA expression of three hepato-specific markers [24,25], albumin, transferrin and Nuclear Factor 4α (HNF 4α), were determined by RT-PCR. As shown in Figure 1, total RNA from primary cultured cells corresponding to the mRNAs of hepato-specific genes (lane 7, HepM) transcripts were amplified. These data demonstrate that our primary cultures are highly enriched in mouse hepatocytes. This result was compared with the amplification of these mRNAs in cell lines derived from human hepatomas (lane 3 and 5, Huh-7 and HepG2, respectively) and human keratinocytes (lane 1, HFK), where no amplification of these mRNAs was observed. Once the presence of hepatocytes in the primary culture was confirmed, we proceeded to analyze the purity of the culture. The constitutively expressed gene 18S rRNA was used as an internal control.

The presence of fibroblasts in the primary culture was determined by Western blot expression of α-smooth muscle actin protein (αSMA). As shown in Figure 1c, only the αSMA was detected in the protein extract of fibroblasts (lane Fb(+)), and it was not present in the extracts generated from the primary culture. This confirms that the primary culture is enriched with hepatocytes and not contaminated with fibroblasts. 

**Figure 2:**
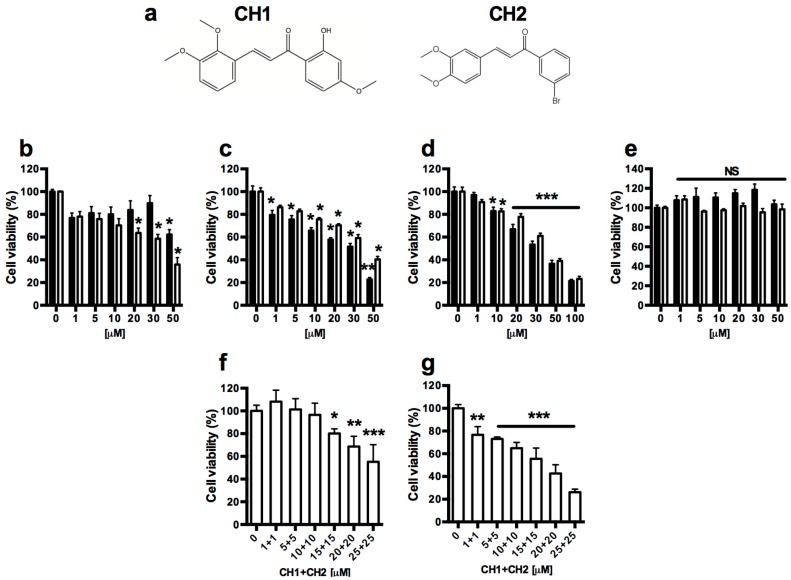
Effect of the chalcones CH1 and CH2 on the growth of human hepatocellular carcinoma (HepG2 and HuH-7) and normal mouse hepatocyte (HepM). Structures of CH1 and CH2 (**a**); HepG2 (**b**,**c**); HuH-7 (**d**) and HepM (**e**) were treated with various concentrations of the compounds CH1 (Black bars) and CH2 (White bars) for 24 (**b**) and 48 (**c**–**e**) h. Chalcones combined (CH1:CH2) were used in HepG2 (**f**) and HuH-7 (**g**) cells for 48 h. Cell viability was measured using the MTT assay. Data are expressed as the mean ± SEM from three independent experiments, each performed in triplicate. Statistical differences were assessed by a one-way ANOVA (Kruskal–Wallis) followed by Dunn’s *post hoc* test. * *p* < 0.05, ** *p* < 0.01, *** *p* < 0.001 compared with the condition 0 μM, NS, Non-significant.

The potential cytotoxic effect of the compounds CH1 and CH2 (Figure 2a) [26,27] were measured in HepG2, HuH-7 and HepM cells. As shown in Figure 2b–d, both chalcones had a cytotoxic effect only in HCC (HuH-7 and HepG2) cells, whereas cytotoxic effects were not observed in mouse hepatocytes (Figure 2e). Dose-response curve experiments were performed using both chalcones. The *IC*_50_ for HepG2 cells is approximately 50 μM at 24 h (Figure 2b) and close to 30 μM (Figure 2c) at 48 h for both chalcones. A similar effect is observed in HuH-7 cells where both chalcones have an approximate *IC*_50_ of 30 μM (Figure 2d) at 48 h. No cytotoxic effects were observed in mouse hepatocytes with the same doses at 48 h (Figure 2e).

Synergistic effects of combination treatment with both chalcones were studied in HepG2 (Figure 2f) and HuH-7 (Figure 2g) cells at 48 h. There is no synergistic effect of both chalcones on HCC cells: the *IC*_50_ (approximately 30 μM) is very similar to that observed with chalcones used separately.

**Figure 3:**
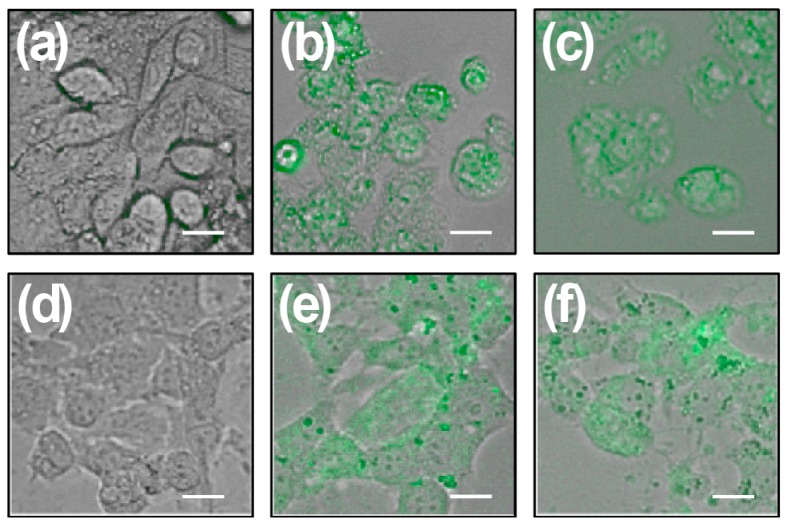
HepG2 and HepM cells exposed to chalcones CH1 and CH2. HepG2 and HepM cells were grown for 24 h, and then treated with each chalcone, independently. Figures show representative phase-contrast images from at least three separates experiments of HepG2 (**a**–**c**) and HepM (**d**–**f**) exposed to vehicle (control) (**a**,**d**); 50 μM CH1 (green) (**b**,**e**); and 50 μM CH2 (green) (**c**,**f**). Bar scale represents 10 μm.

HepG2 cells and mouse hepatocytes show similar intracellular accumulation of chalcones, whereas cells treated with the vehicle did not exhibit green fluorescence (Figure 3a,d). Cells treated with chalcones for 24 and 48 h show intracellular green marks that are attributed to the presence of the chalcone CH1 (Figure 3b,e) and CH2 (Figure 3c,f). These results demonstrate that both chalcones enter the cells independently.

### 2.2. Both Chalcones Induces Apoptosis in Human Hepatoma through Caspase-Dependent Pathways

**Figure 4:**
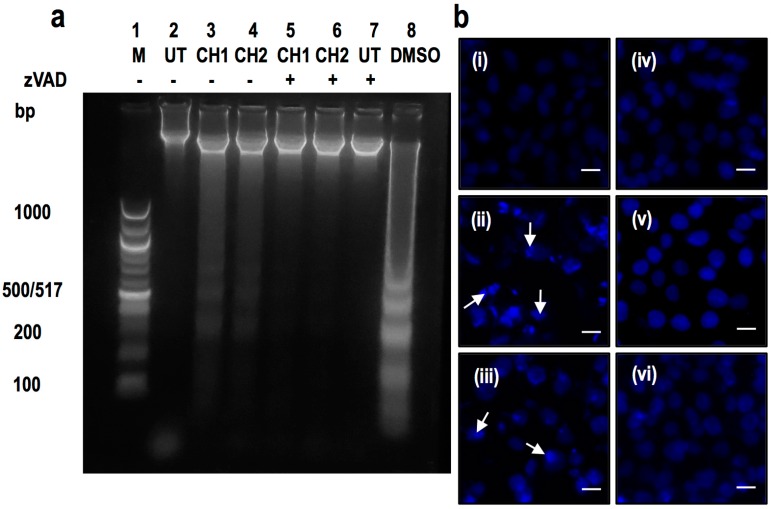
Chalcones induced laddering and nuclear condensation of HepG2 cells a process that requires caspase activity. (**a**) Cells were treated with CH1 and CH2 (50 μM) for 24 h and preincubated with zVAD (50 μM) for 2 h (case +), when the cells were incubated in the absence of inhibitor (case -), typical laddering indicative of apoptosis was observed. DMSO was used as control positive of laddering; (**b**) HepG2 (**i**–**iii**) and HepM (**iv**–**vi**) cells were treated with vehicle (**i**,**iv**), CH1 (**ii**,**v**) and CH2 (**iii**,**vi**) for 24 h. Cells were stained with DAPI (blue) and the arrow indicated nuclear condensation. Bar scale represents 10 μm.

**Figure 5:**
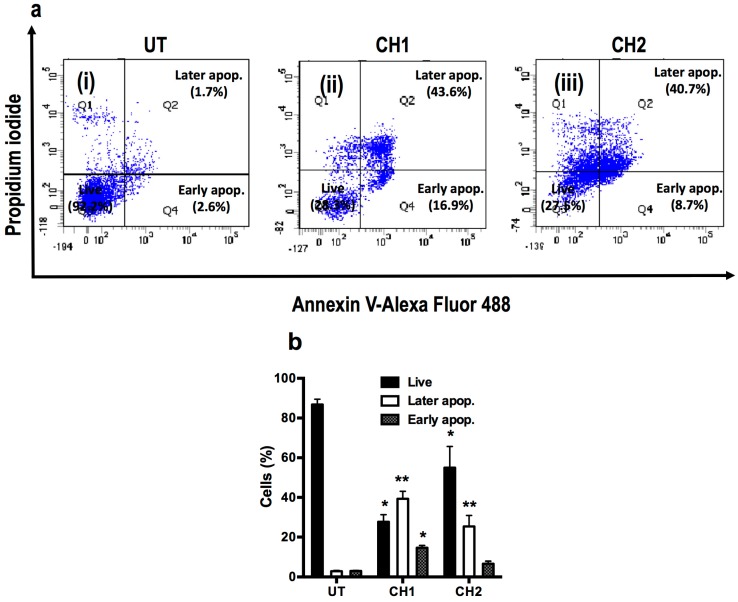
Chalcone-induced apoptosis in HepG2 cells as assayed by flow cytometry. (**a**) HepG2 cells were treated with CH1 and CH2 (50 μM) for 24 h. The cells were then harvested and stained with Annexin V and PI and flow cytometric analysis was performed to analyze the apoptosis; (**b**) Summary of the apoptosis data in histogram form. Data are expressed as the mean ± standard error of the mean (SEM) from at least three independent experiments. * *p* < 0.05 *vs.* the untreated (UT) group; ** *p* < 0.01 *vs.* the untreated group.

The presence of a laddered DNA fragmentation pattern, typical of apoptotic cells, was evaluated in HepG2 after 24 h of exposure to both chalcones (Figure 4). The chalcone CH1 and CH2 induced DNA fragmentation at a dose of 50 μM (Figure 4a, lane 3 and 4), whereas the addition of a caspase inhibitor (zVAD) blocked the DNA fragmentation induced by both chalcones (Figure 4a, lane 5 and 6). Cells treated with DMSO, an inducer of apoptosis, provided a positive control for ladder formation [28] (Figure 4a, lane 8). Cells stained with 4′-6-diamidino-2-phenylindole (DAPI) showed high nuclear condensation in HepG2 cells treated for 24 h with CH1 (Figure 4b(ii)) and CH2 (Figure 4b(iii)). HepM cells did not show nuclear condensation with either chalcone (Figure 4b(v,vi)) and did not present differences from the control cells (Figure 4b(i,iii)). According to the results obtained with ladder fragmentation and nuclear condensation, the next experiment was performed to quantify apoptotic cells. Flow cytometry was used to measure Annexin V-FITC and PI stained cells, which confirmed the induction of apoptosis in human hepatoma cells with both chalcones (Figure 5). As expected, both compounds increased the number of apoptotic cells. 1.7% of human hepatoma cells that were treated with vehicle were in the late stage of apoptosis and 2.6% were early in apoptosis (Figure 4a(i)), whereas cells that were treated for 24 h with the chalcones CH1 and CH2 exhibited an increase in late apoptosis of 43.6% and 40.7%, respectively (Figure 5a(ii,iii)). Moreover, CH1 and CH2 exhibited an increase of early apoptotic cells of 16.9% and 8.7%, respectively (Figure 5a(ii,iii)).

As shown in Figure 5b, significant differences were found between populations of live (27.7% ± 3.6%, 55.0% ± 10.7%; *p* < 0.05) and apoptotic (39.3% ± 3.8%, 35.4% ± 5.5%; *p* < 0.01) human hepatoma cells treated with CH1 and CH2, respectively. However, only treatment with CH1 resulted in significant differences in early apoptosis (14.6% ± 1.2%; *p* < 0.05). These results demonstrated that both chalcones stimulate human hepatoma cells to initiate apoptosis, a process that requires caspase activity.

### 2.3. Chalcones Induces Cell Death through an Intrinsic Apoptotic Pathway

**Figure 6:**
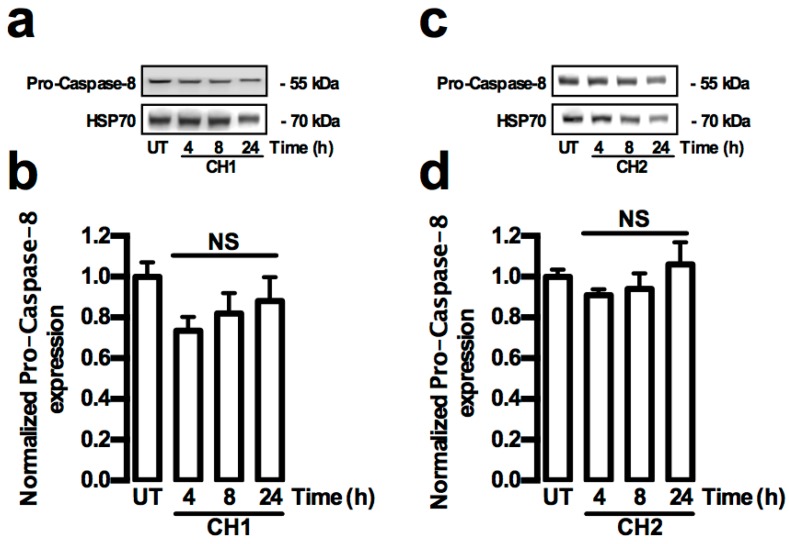
Chalcones do not induce caspase extrinsic pathway in HepG2 cells. (**a**–**d**) Cells were treated with CH1 and CH2 (50 μM, each) and protein expression was analyzed. (**a**,**c**) Representative images from Western blot experiments performed for the detection of caspase-8; (**b**,**d**) Densitometric analyses of the experiments shown in (**a**,**c**), respectively. Protein levels were normalized against HSP70 and data are expressed relative to the UT (untreated) condition. Data are expressed as the mean ± standard error of the mean (SEM) from at least three independent experiments. Statistical differences were assessed by a one-way ANOVA (Kruskal–Wallis) followed by Dunn’s *post hoc* test. NS: Not significant.

**Figure 7:**
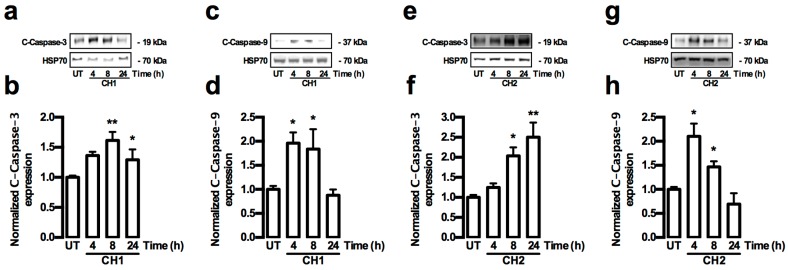
Chalcones-induced caspase intrinsic pathway in HepG2 cells. (**a**–**h**) Cells were treated with CH1 and CH2 (50 μM, each) and protein expression was analyzed. (**a**,**c**,**e**,**g**) Representative images from Western blot experiments performed for the detection of C-caspase-9 and C-caspase-3; (**b**,**d**,**f**,**h**) Densitometric analyses of the experiments shown in (**a**,**c**,**e**,**g**), respectively. Protein levels were normalized against HSP70 and data are expressed relative to the UT (untreated) condition. Data are expressed as the mean ± standard error of the mean (SEM) from at least three independent experiments. Statistical differences were assessed by a one-way ANOVA (Kruskal–Wallis) followed by Dunn’s *post hoc* test. * *p* < 0.05; ** *p* < 0.01 *vs.* UT group.

**Figure 8:**
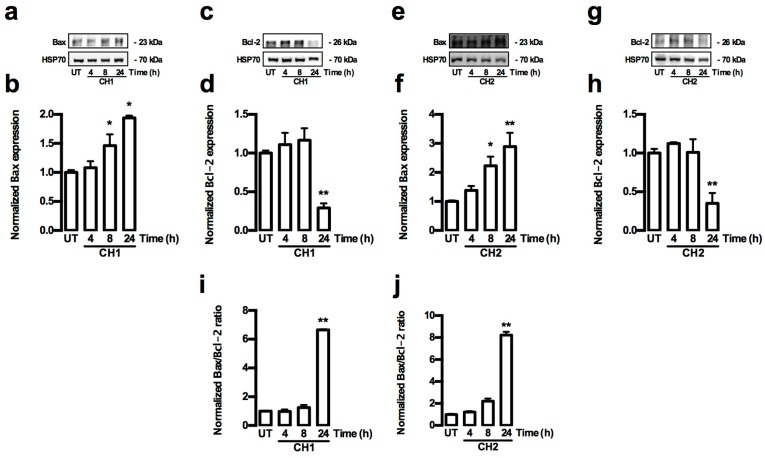
Chalcones-induced increase ratio Bax/Bcl-2 in HepG2 cells. (**a**–**h**) Cells were treated with CH1 and CH2 (50 μM, each) and protein expression was analyzed; (**a**,**c**,**e**,**g**) Representative images from Western blot experiments performed for the detection of Bax and Bcl-2; (**b**,**d**,**f**,**h**) Densitometric analyses of the experiments shown in (**a**,**c**,**e**,**g**), respectively. (**i**,**j**) the data were presented in the bar graphs as Bax/Bcl-2 ratio. Protein levels were normalized against HSP70 and data were expressed relative to the UT (untreated) condition. Data are expressed as the mean ± standard error of the mean (SEM) from at least three independent experiments. Statistical differences were assessed by a one-way ANOVA (Kruskal–Wallis) followed by Dunn’s *post hoc* test. * *p* < 0.05; ** *p* < 0.01 *vs.* UT group.

Caspase proteins play a major role in the activation of apoptosis. For this reason, both extrinsic and intrinsic activation pathways by caspases were analyzed in HepG2 cells treated with both chalcones. The extrinsic and intrinsic pathways of caspase activation were determined by Western blot analysis of the caspase-3, -8 and -9 activities, and also by the pro-apoptotic protein Bax and anti-apoptotic protein Bcl-2. The activity of the caspase 8 protein (extrinsic pathway) in HepG2 cells did not show any increase in the presence of both chalcones (Figure 6a–d). The total amount of the cleaved caspase-9 (C-caspase-9) protein (intrinsic pathway) increased significantly (*p* < 0.05) at 4 and 8 h (Figure 7a,b), whereas the total cleaved caspase-3 (C-caspase-3) protein level significantly increased (*p* < 0.01) at 8 h in HepG2 cells treated with CH1 (Figure 7c,d). Similar results were observed after treatment with CH2, where the C-caspase-9 protein (intrinsic pathway) total significantly increased (*p* < 0.05) at 4 h (Figure 7e,f), whereas the C-caspase-3 protein total significant increased (*p* < 0.01) at 8 and 24 h in HepG2 cells treated with CH2 (Figure 7g,h). The data coincided with a significant increase in the Bax protein (*p* < 0.05) at 8 and 24 h (Figure 8a,b,e,f) and a significant decrease in the Bcl-2 protein (*p* < 0.01) at 24 h (Figure 8c,d,g,h) in HepG2 cells treated with CH1 and CH2, respectively. Densitometric analysis revealed significant increase of Bax/Bcl-2 ratio at 24 h with CH1 and 8 and 24 h with CH2 (Figure 8i,j). These results demonstrated that both chalcones induce apoptosis through the intrinsic caspase pathway. 

**Figure 9:**
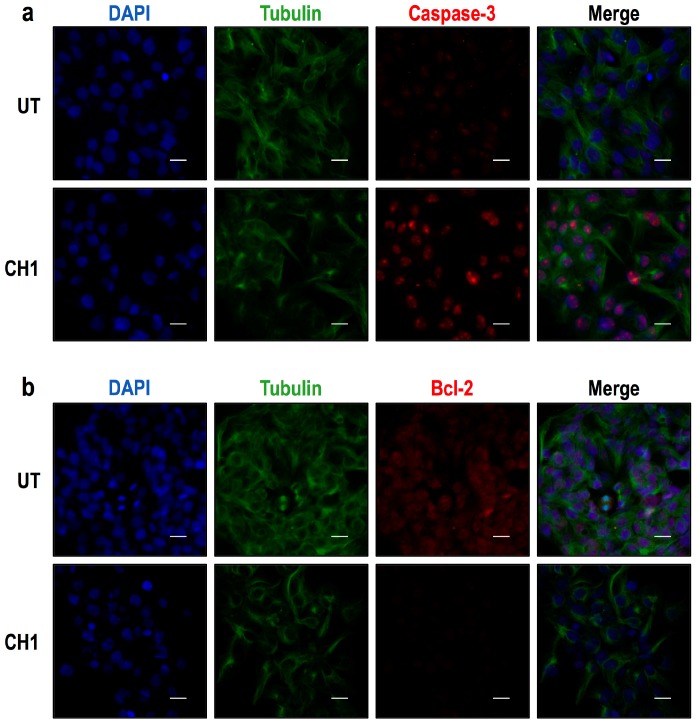
Protein expression of caspase-3 and Bcl-2 in HepG2 cells as shown by immunocytochemical analysis. HepG2 cells were treated with 50 μM of CH1 for 24 h, and then immunocytochemical analyses were performed as described in Materials and Methods. Cells were fixed and immunostained with anti-tubulin antibody (green), anti-caspase-3 (red) and cell nuclei were counterstained with DAPI reagent (blue). (**a**) The results revealed that CH1 increased caspase-3 expression in HepG2 cells; (**b**) CH1 was shown to decrease Bcl-2 expression in HerpG2 cells. These findings also suggest that CH1 induces the apoptosis of HepG2 cells *in vitro*. Tubulin was used as expression control. Bar scale represents 20 μm.

**Figure 10:**
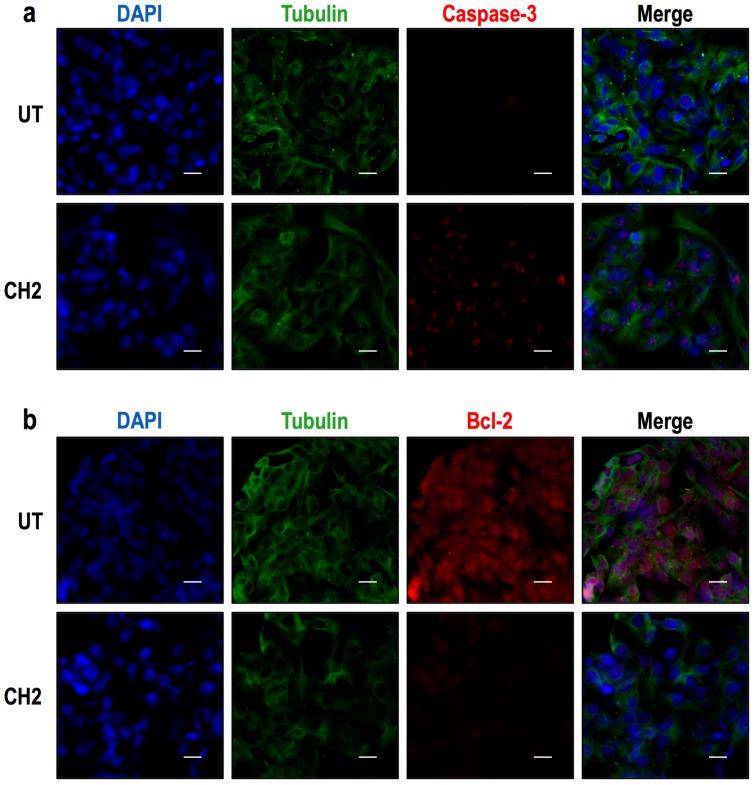
Protein expression of caspase-3 and Bcl-2 in HepG2 cells as shown by immunocytochemical analysis. Cells were treated with 50 μM of CH2 for 24 h, and then immunocytochemical analyses were performed as described in Materials and Methods. Cells were fixed and immunostained with anti-tubulin antibody (green), anti-caspase-3 (red) and cell nuclei were counterstained with DAPI reagent (blue). (**a**) The results revealed that CH2 increased caspase-3 expression in HepG2 cells; (**b**) CH2 was shown to decrease Bcl-2 expression in HerpG2 cells. These findings also suggest that CH2 induces the apoptosis of HepG2 cells *in vitro*. Tubulin was used as expression control. Bar scale represents 20 μm.

Immunocytochemistry analyses were also performed. HepG2 cells were treated with CH1 (50 μM) for 24 h (Figure 8), and the cells were then fixed with 3.7% paraformaldehyde prior to the detection of caspase-3 and Bcl-2 protein expression. The results revealed that CH1 increased caspase-3 expression (Figure 9a) and decreased Bcl-2 expression (Figure 9b) in HepG2 cells. Similarly, the CH2 compound increased caspase-3 expression (Figure 10a) and decrease Bcl-2 expression (Figure 10b). These findings also suggest that both chalcones induce the apoptosis of HepG2 *in vitro*.

### 2.4. Chalcones Lead to Increased ROS

**Figure 11:**
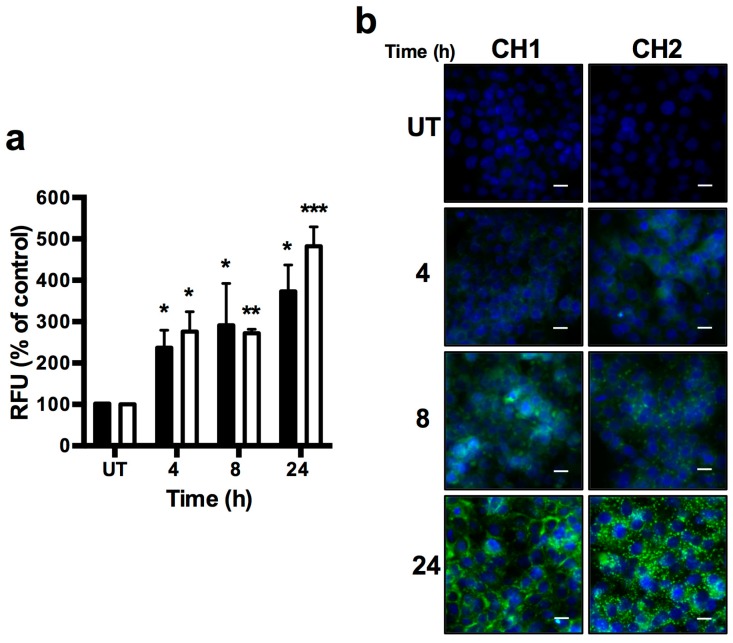
Chalcone effects on the HepG2 cells; Intercelular reactive oxygen species generation. CH1 and CH2 enhanced cellular ROS level. Cells were exposed to CH1 and CH2 at 50 μM for 4, 8 and 24 h. (**a**) Stained cells with DCFDA and analyzed by fluorescence in a plate reader (Tecan infinite^®^ m200pro, Grodig, Austria); (**b**) Stained cells with DAPI (blue) and DCFDA (green), and analyzed under fluorescent microscopy EVOS^®^ FLoid^®^ cell (Life Tehnologies, CA, USA), Black bars represents CH1 and White bars represents CH2. Data are expressed as the mean ± standard error of the mean (SEM) from three independent experiments, each performed in triplicate. Statistical differences were assessed by a one-way ANOVA (Kruskal–Wallis) followed by Dunn’s *post hoc* test. * *p* < 0.05; ** *p* < 0.01; *** *p* < 0.001 *vs.* Untreated (UT) group. Bar scale represents 20 μm.

To analyze the levels of hydrogen peroxide in HepG2 cells treated with both chalcones, we used a fluorescent probe, DCFDA, to detect ROS. Increased ROS levels were observed in HepG2 cells treated with both compounds, together with an early and late rise in ROS. As Figure 11a shows, 4 to 24 h after treatment, an increase in mean of RFU of cells treated with both chalcones were observed; RFU increased to 237% for CH1 and 276% for CH2 at 4 h, 291% for CH1 and 272% for CH2 at 8 h, and 373% for CH1 and 482% for CH2 at 24 h in comparison to the untreated group. Consistent with the cytosolic fraction observations, we also observed an increase in the ROS levels by microscopy in HepG2 cells exposed to both chalcones (Figure 11b).

## 3. Discussion

In recent decades, many efforts have been made to develop drugs for cancer treatment based on the ability to block cell proliferation and/or induce apoptosis [29]. Apoptosis is a form of physiological cell death that is essential for normal tissue development and homeostasis [30,31]. This process can clearly be identified by characteristic changes in the cells (*i.e*., caspase activation, DNA fragmentation, cell fragmentation through the formation of “apoptotic bodies”) [32]. Chalcones, considered as the precursors of many biologically important heterocycles (*i.e.*, flavonoids, isoflavonoids, benzothiazepine, pyrazolines and 1,4-diketones). Thus, the synthesis of new chalcone derivatives is of great interest for medicinal chemists, especially because Chalcones have demonstrated to be very active against different cancer cell lines [33,34]. Human hepatomas are a major contributor to cancer deaths worldwide. Many efforts have been made to reduce the use of chemotherapy and introduce alternative chemicals that could help to improve the patient’s quality of life. Chalcones have been widely studied due to their capacity to generate anti-tumor effects in a variety of cancers [33,34,35,36,37], and great efforts have been made to use chalcones to improve treatments for hepatic cancer [19,38,39,40].

The aim of our study is to identify novel compounds (*i.e.*, chalcones) that activate apoptosis in hepatome and have low (or no) toxicity against liver cells. However, to consider chalcones as an anti-tumoral, the present study needs to be verified by further extensive *in vivo* testing.

We found that both chalcones, CH1 and CH2 generate cytotoxicity in human hepatome cells (HepG2) but not in normal hepatic cells (HepM). The induction of apoptosis in HepG2 cells is caspase-dependent and acts through the intrinsic pathway to generate fragmentation and nuclear condensation and increased levels of ROS.

Other researchers have also reported the use of chalcones for the potential treatment of HCC [41,42], but its effect was not demonstrated in normal hepatic cells. Many efforts have been made to synthesize compounds that destroy only the hepatoma, causing no damage to normal hepatic cells [42,43]. In this work, for the first time, we document the application of two chalcones in hepatic tumor cells. Our results showed that CH1 and CH2 have some effect on hepatic tumor cells and have no cytotoxic effect on normal hepatic cells (Figure 2). We have previously reported that chalcones exerts a cytotoxic effects in HepG2 cells [19] (*IC*_50_ > 100 μM). However, researchers have recently reported that another group of chalcones have *IC*_50_ similar to CH1 and CH2 in HepG2 cells [44] (*IC*_50_ < 50 μM). Although the *IC*_50_ of these chalcones is high, other chalcones have similar cytotoxic effect in a variety of tumors [45,46,47,48]. Both chalcones (CH1 and CH2) tested induced cytotoxicity through caspase-dependent apoptosis (Figure 3 and Figure 4). As previously documented, chalcones can activate intrinsic and extrinsic pathways [29,49]. Taking this evidence into account, the activity of caspase-8 (Figure 5a–d), caspase-9 (Figure 6a,b,e,f) and caspase-3 (Figure 6c,d,g,h) were analyzed. In our case, only caspase-3 and caspase-9 were activated by both chalcones, demonstrating that the intrinsic pathway is crucial to induce apoptosis in HepG2 cells. To determine the location and distribution of caspase-3 (proapoptotic) and Bcl-2 (antiapoptotic) we performed immunocytochemistry assays. The results showed that both chalcones increased the presence of caspase-3 and also demonstrated that they are located mainly in the nucleus of HepG2 cells (Figure 8 and Figure 9). This effect has already been observed by other researchers, indicating that caspase-3 has a major role in the progression of apoptosis [50]. Furthermore, our studies showed that both chalcones resulted in an obvious decrease in the Bcl-2 protein expression and a notable increase in Bax protein production in the HepG2 cells (Figure 6 and Figure 7). The Bcl-2 protein family includes proapoptotic members such as bax, bak, bad, bcl-xs, bid, bik, bim, and hrk, and antiapoptotic members such as bcl-2, bcl-xl, bcl-w, bfl-1, and mcl-1 [51,52,53]. Researchers have suggested that changes in ratio of Bax to Bcl-2 may contribute to the caspase-3 activation and the modulation of renal and neuronal apoptosis in rats [54,55]. Moreover, in HepG2 cells, it was demonstrated that the compound Xanthorrhizol decreased the regulation of the Bcl-2 protein but did not affect the Bax protein while activating caspase-3 and caspase-9. Xanthorrhizol exerts anti-proliferative effects on HepG2 cells by inducing apoptosis via the mitochondrial pathway [56]. The mitochondria are primarily responsible for the generation of intracellular ROS [57]. Increased ROS levels alter cellular function through the oxidation of amino acids in proteins, lipids, and DNA damage, which triggers apoptotic processes [58]. Our results show a progressive increase in ROS from 4 to 24 h with both chalcones (Figure 10a,b). Other research groups have reported increased ROS in different cell lines stimulated by chalcones [41,59]; and the antioxidant effect of chalcones has also been reported [60,61,62,63]. 

## 4. Materials and Methods

### 4.1. Synthesis of Chalcone

We have previously described the synthesis of the following compounds: 2,3,4′-trimethoxy-2′-hydroxy-chalcone (CH1) [26] which was obtained as orange crystals (58%), mp. 129–130 °C; and 3′-bromo-3,4-dimethoxy-chalcone (CH2) [27] obtained as yellow crystals (73%), mp. 117–120 °C (Figure 1a).

### 4.2. Cell Culture

Human hepatocellular carcinoma HepG2 cells (American Type Culture Collection HB-8065) and Huh-7 were grown in monolayer culture in Dulbecco’s modified Eagle Medium (DMEM) with 10% fetal bovine serum (FBS) (Gibco, NY, USA) and antibiotic-antimycotic (Gibco, NY, USA) at 37 °C in a humidified 5% CO_2_ incubator.

Primary culture of mouse hepatocytes (HepM) were isolated from male BALB/C mice (three weeks old) using a modification of the perfusion and enzymatic digestion protocol as previously described [64]. Briefly, the liver was first perfused with liver perfusion buffer I 0.9% NaCl (Merck, Darmstadt, Germany), 100 μg/ mL gentamicin (Gibco, Grand Island, NY, USA), 2× antibiotic-antimycotic (Gibco, NY, USA), 0.2 mM EGTA (Sigma, USA) to disrupt hepatocyte desmosomes. In order to separate hepatocytes from the extracellular matrix, a second perfusion was performed with liver perfusion buffer II 0.5% collagenase I, II, IV (Gibco, Grand Island, NY, USA), 0.25% hyaluronidase (Sigma), 0.1% Dispase (Gibco), 0.025% DNase I (Gibco), 0.1% BSA (Rockland, Limerick, PA, USA), 2× antibiotic-antimycotic (Gibco, Grand Island, NY, USA), 1 mM CaCl_2_ (Sigma) in DMEM GlutaMaxTM (Gibco, Grand Island, NY, USA). The liver was then removed and cut in fine pieces, followed by three washes in PBS and incubated in liver perfusion buffer II at 250 RPM for 1 h at 37 °C. Then, the cells were centrifuged at 300× *g* for 5 min, washed in PBS (as described before) and incubated with 4× trypsin at 250 RPM for 15 min at 37 °C. The cells were centrifuged and washed (as described before) and incubated in lysis buffer (8.3 g NH_4_Cl, 1.0 g KHCO_3_, 5% EDTA) at 250 RPM for 10 min at 37 °C. Finally the hepatocytes were plated and cultured on collagen-coated tissue culture plate to 70% confluent and maintained in hepatocyte medium DMEM GlutaMax™ (Gibco, Grand Island, NY, USA): DMEM/F12 (Gibco, Grand Island, NY, USA) (1:1) supplemented with 1× Insulin-transferrin-selenium (Gibco, Grand Island, NY, USA), 5 μg/mL hydrocortisone (Sigma), 10% FBS (Biological Industries, Kibbutz Beit-Haemek, Israel), 2× Antibiotic-Antimycotic (Gibco, Grand Island, NY, USA) and 20 mM HEPES (Gibco, Grand Island, NY, USA) at 37 °C in 5% CO_2_. HepG2 and HepM cells were treated with chalcones CH1 and CH2 diluted in Milli-Q water. The Milli-Q water was also used in the untreated control (UT).

### 4.3. Ethics Approval

This research was performed according to the “Guides for the Care and Use of Laboratory Animals” [65]. Mice were kept in cages with controlled light conditions (12 h light/darkness) and given food and water *ad libitum*. The use of these animals was approved by the “bioethics and biosafety committee” of Fundación Ciencia para la Vida. The maintenance of the animals was performed in the animal facility of Fundación Ciencia para la Vida. During the whole course of animal experiments, all efforts were made to minimize suffering.

### 4.4. MTT Assay

HepG2 cells were seeded in a 96-well plate at an initial density of 5 × 103 cells/well. Twenty-four hours later, the cells were treated with control (Milli-Q water) or various concentrations of chalcone for 24 and 48 h. The MTT (3-(4,5-dimethyl-2-thiazolyl)-2,5-diphenyl-2*H*-tetrazolium bromide) method was used as we previously described [66]. The absorbance of the dissolved formazan crystals was measured at 540 nm using a microplate reader (Tecan infinite^®^ f50, Grodig, Austria).

### 4.5. Western Blot 

Western blot method was used as we previously described [67]. For a detailed list of antibodies used, see Table S1.

### 4.6. Fluorescence Microscopy

Fluorescence microscopy method was used as we previously described [67]. For a detailed list of antibodies used, see Table S2. Fluorescent cells were analyzed using the microscopy EVOS^®^ FLoid^®^ cell (Life Technologies, Carlsbad, CA, USA).

### 4.7. Determination of Intracellular ROS

2,7-Dichlorodihydrofluorescein diacetate (DCFH-DA), method was used as we previously described [68]. DCF fluorescence was detected by a fluorescence plate reader (Tecan infinite^®^ m200pro). ROS levels were expressed as the relative fluorescence unity’s (RFU) of DCF. Fluorescent cells were analyzed using the EVOS^®^ FLoid^®^ cell (Life Technologies, Carlsbad, CA, USA).

### 4.8. Annexin-V Apoptosis Assay

Apoptosis was measured through flow cytometry. Detection of phosphatidylserine expression on early apoptotic cells using fluorescein labeled Annexin—V using “Alexa Fluor 488 Annexin V/Dead Cell Apoptosis” Kit, the protocol was carried out as described by the manufacturer’s instructions (Invitrogen, Carlsbad, CA; USA). The cells were analyzed in a flow cytometer model BD FACS Canto II and 10,000 events were analyzed by sample. 

### 4.9. DNA Laddering Experiments

HepG2 cells were seeded in 6-well plates at a density of 105 per well and exposed to 50 μM of chalcone for 24 h. DNA laddering method was used as we previously described [69].

### 4.10. RT-PCR Amplification

Total RNA from cells was extracted with Trizol (Invitrogen) as described before [70]. To eliminate DNA contamination, RNA preparations were treated with TURBO DNA-freeTM (Ambion, Austin, TX, USA) according to the manufacturer’s instructions. Reverse transcription was carried out with 100 ng of freshly prepared RNA, 50 ng of random hexamers primers, and 100 units of reverse transcriptase (Moloney murine leukemia virus, Invitrogen) and 20 units of RiboLock (Thermo Scientific, Waltham, MA, USA) as described before [70]. The PCR-amplified were made in a volume of 50 μL containing 1× Go Taq Flexi Buffer, 1.5 mM MgCl_2_, dNTPs (0.4 μM), 2.0 μL cDNA, 1.0 μM of each primer and 2.5 units of Go Taq Flexi DNA Polymerase (Invitrogen). Standard conditions for amplification were as follows: initial denaturation step at 94 °C for 5 min, then 30 cycles at 94 °C for 1 min, then kept at 56 °C for 1 min, 72 °C for 1 min, followed by a final extension step of 72 °C for 5 min. The primers used to amplify the genes products of Transferrin, Albumin and HNF 4α were designed to recognize the Human and Mouse mRNA sequence. The primers used for amplification were the following: Transferrin mRNA, Transferrin (r) (5′-GTGCTTGACAAAGGCCACA) and Transferrin (f) (5′-AGGCAAGAAGTCCTGCCA), Albumin mRNA, Albumin (r) (5′-TGCCCAGGAAGACATCCTT) and Albumin (f) (5′-AAAGCATGGGCAGTAGCTC); HNF 4α mRNA, HNF 4α (r) (5′-ACCTGCTCTACCAGCCAGAA) and HNF 4α (f) (5′-GCAGGGTCTAGAAGGCTGTG). Amplified DNA fragments were analyzed by electrophoresis. 

### 4.11. Data Analysis

In all experiments, replicates were made and the data presented represent the average ± standard error (SEM) of at least three independent experiments performed in triplicate. The comparisons were conducted using one-way analysis of variance (ANOVA) (Kruskal–Wallis) followed by Dunn’s *post hoc* test and considered significant at *p* < 0.05.

## 5. Conclusions

**Figure 12:**
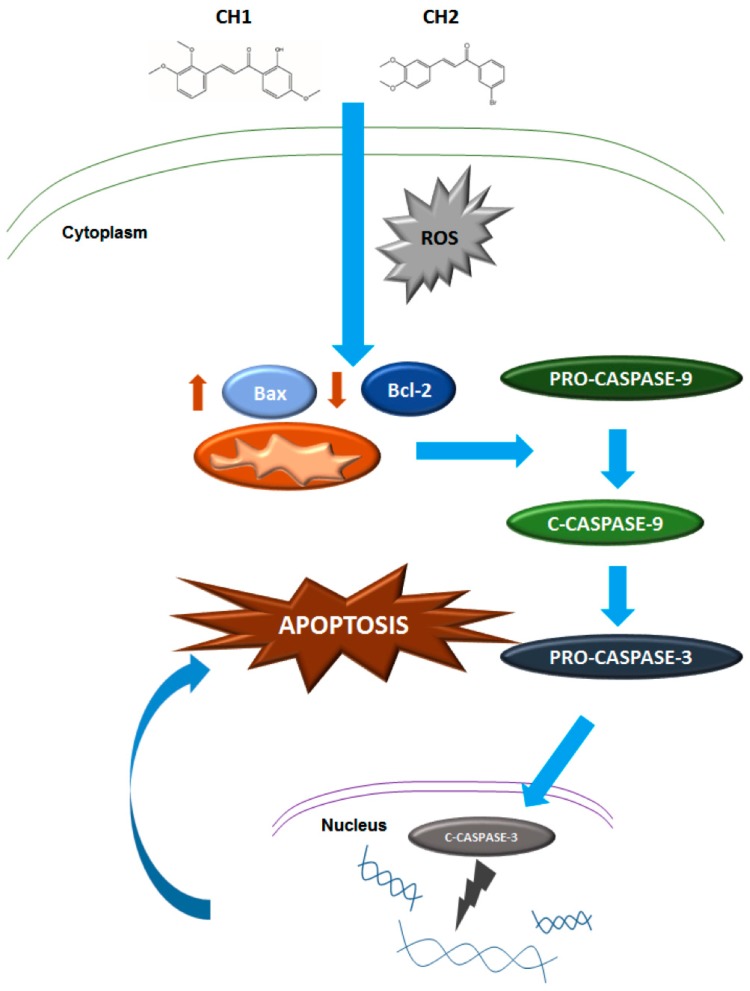
Proposed model for Chalcone-mediated apoptosis in human hepatoma cells. Solid arrow represent activation.

Our results demonstrate that both chalcones have a cytotoxic effect on human hepatoma through the induction of caspase-dependent apoptosis. These chalcones activate intracellular ROS levels and could be responsible for the activation of programmed death, through the intrinsic mitochondrial pathway mediated by Bax/Bcl-2 and the modulation of caspase-9 and caspase-3 (Figure 12). Furthermore, neither chalcones have cytotoxic effects on normal mouse hepatocytes. This would allow the potential use of chalcones as a selective drug to target only tumor cells. Further experiments *in vivo* are needed to verify the possible application of chalcones as selective drugs for the treatment of cancer.

## Supplementary Materials

Supplementary materials can be found at http://www.mdpi.com/1422-0067/17/2/260/s1.
