# Supplementary material for: Chalcone-Induced Apoptosis through Caspase-Dependent Intrinsic Pathways in Human Hepatocellular Carcinoma Cells
Source: Int J Mol Sci. 2016 Feb 22;17(2):260. doi: 10.3390/ijms17020260 (PMC4783989; doi:10.3390/ijms17020260)
Supplement: Supplementary file 1 [file ijms-17-00260-s001.pdf]

# Supplementary Materials: Chalcone-Induced Apoptosis through Caspase-Dependent Intrinsic Pathways in Human Hepatocellular Carcinoma Cells

**Rodrigo Ramirez-Tagle, Carlos A. Escobar, Valentina Romero, Ignacio Montorfano, Ricardo Armisén, Vincenzo Borgna, Emanuel Jeldes, Luis Pizarro, Felipe Simon and Cesar Echeverria**

**Table S1.** Primary and secondary antibodies used in Western blot experiments.

| Primary Ab | Dilution | Source | Incubation Time | Incubation Temperature | Band |
|---|---|---|---|---|---|
| Bax | 1:500 | Rabbit | 2 h | RT | Santa Cruz, CA, USA |
| Bcl-2 | 1:500 | Rabbit | 2 h | RT | Santa Cruz, CA, USA |
| Caspase-3 | 1:1000 | Rabbit | 2 h | RT | Cell Signaling, MA, USA |
| Caspase-8 | 1:1000 | Mouse | 2 h | RT | Santa Cruz, CA, USA |
| Caspase-9 | 1:1000 | Mouse | 2 h | RT | Cell Signaling, MA, USA |
| HSP70 | 1:5000 | Mouse | 2 h | RT | Origene, MD, USA |
| **Secondary Ab** | **Dilution** | **Source** | **Incubation Time** | **Incubation Temperature** | **Band** |
| Anti-Mouse HRP | 1:5000 | Goat | 2 h | RT | Millipore, MA, USA |
| Anti-Rabbit HRP | 1:5000 | Goat | 2 h | RT | Millipore, MA, USA |

Ab: antibody; RT: room temperature; ON: overnight.

**Table S2.** Primary and secondary antibodies used in immunocytochemistry.

| Primary Ab | Dilution | Source | Incubation Time | Incubation Temperature | Band |
|---|---|---|---|---|---|
| Bcl-2 | 1:100 | Rabbit | ON | 4 °C | Santa Cruz, CA, USA |
| Caspase-3 | 1:100 | Rabbit | ON | 4 °C | Cell Signaling, MA, USA |
| $\alpha$-Tubulin | 1:500 | Mouse | ON | 4 °C | Sigma-Aldrich, Mo, USA |
| **Secondary Ab** | **Dilution** | **Source** | **Incubation Time** | **Incubation Temperature** | **Band** |
| Anti-Rabbit Alexa-594 | 1:250 | Goat | ON | 4 °C | Invitrogen, CA, USA |
| Anti-Mouse Alexa-488 | 1:250 | Goat | ON | 4 °C | Invitrogen, CA, USA |

Ab: antibody; RT: room temperature; ON: overnight.
